# A Study on the Microstructure and Mechanical Properties of Portland Cement Incorporating Aluminosilicate Waste

**DOI:** 10.3390/ma17020354

**Published:** 2024-01-10

**Authors:** Valentin Antonovič, Donatas Sikarskas, Renata Boris, Andrius Kudžma, Jurgita Malaiškienė, Rimvydas Stonys

**Affiliations:** Laboratory of Composite Materials, Institute of Building Materials, Vilnius Gediminas Technical University, 10223 Vilnius, Lithuaniarenata.boris@vilniustech.lt (R.B.);

**Keywords:** aluminosilicate waste, metakaolin, fluid catalytic cracking catalyst, microstructure, compressive strength, density, binder

## Abstract

The influence of aluminosilicate pozzolanic waste, specifically spent fluid catalytic cracking waste (FCCW) and metakaolin waste (MK) from the expanded glass industry, on the properties of hardened Portland cement paste were analysed. The study involved replacing part of cement with FCCW and MK and observing their impact on the hydration, microstructure, density, and compressive strength of hardened cement paste. Various analysis methods were employed, including X-ray diffraction (XRD), thermogravimetric analysis (TG), and scanning electron microscopy (SEM), to understand the changes in the structure of the hardened cement paste during hydration. The findings revealed that FCCW tends to accelerate the cement hydration process due to its high surface area and pozzolanic activity. Notably, the formation of portlandite crystals was observed on FCCW particle surfaces in a specific direction. These crystals appeared smaller and developed in different directions in compositions containing a composite binder with mixture of FCCW and MK in a ratio 1:1. This could be influenced by pozzolanic reactions activated by fine particles of MK and the formation of calcium silicate hydrates (C-S-H) and calcium alumino silicate hydrates (C-A-S-H) in the presence of portlandite. The XRD and TG results indicated that the specimens containing a composite binder exhibited the least amount of portlandite. The compressive strength of these specimens increased compared to the control specimens, although the amount of cement was 9% lower.

## 1. Introduction

The main goal of several studies [1,2,3] has been to adopt alternative binder systems with advantages such as cost-effectiveness and environmental friendliness. It is important to use materials that can also improve the mechanical properties of cementitious materials without the need to increase the amount of cement due to the high CO_2_ emissions in cement manufacturing and the higher cost of the product.

Various pozzolanic additives can be used to accelerate cement hydration and to improve the structure, mechanical properties, and other characteristics of hardened cement paste. These pozzolanic additives include silicates or aluminates which react with calcium hydroxide (CH) and produce calcium silicate hydrates (CSH) and calcium aluminosilicate hydrates (CASH) [4,5]. The incorporation of industrial waste such as silica fumes, coal industry fly ash, blast furnace slag, rice husk ash, spent catalytic cracking catalysts (FCCW) from oil refineries, and other types of waste, in addition to pozzolanic materials of natural origin, such as volcanic rocks and clays, could make an even more significant contribution to the scaling of the circular economy and addressing climate change [6,7,8,9].

More than 800 thousand tonnes of aluminosilicate waste such as FCCW, which is composed of approximately 40% Al_2_O_3_ and 50% SiO_2_, are generated worldwide per year [10,11]. Many studies have shown that FCCW improves the properties of different types of cement. Therefore, research on FCCW recovery in cementitious materials is ongoing [2,12,13,14,15]. FCCW has already been shown to accelerate cement hydration in the initial stage [16,17], and replacing 5–10% cement with FCCW promotes cement hydration. FCCW, like other pozzolanic additives, improves the mechanical properties of cementitious materials. The results obtained in different studies [18,19,20] show that the maximum strength of the cementitious material was obtained when the FCCW content was 10%. Based on the results described by the authors of [21], 15–20% of cement can be replaced with FCCW without compromising the performance of cement mortar.

Another aluminosilicate material, metakaolin, is made from kaolin fired at temperatures 550–850 °C. Adding the right amount of this pozzolanic additive into the mix can effectively improve the physical and mechanical properties of concrete [22,23,24]. The authors of [25,26] state that 10–15% is the optimal content of metakaolin in cement mixes. If a cementitious material contains a high content of metakaolin, its particles agglomerate, and the CSH formation process is nearly stagnant during the early period of cement hydration [27]. The pozzolanic effect of metakaolin on the properties of concrete and mortars depends on its type. It was also found in [28] that high-quality metakaolin, which contains more than 90% Al_2_O_3_·SiO_2_, accelerates cement hydration. Conversely, lower-grade metakaolin (31–36% Al_2_O_3_·SiO_2_) slows cement hydration [29].

Studies have shown that by using several pozzolanic additives, each with a different effect on the cement hydration mechanism, new positive effects on cement properties can be achieved. Soriano et al. [30] found that using less active fly ash together with FCCW, which has significantly higher pozzolanic activity, can increase fly ash efficiency. Such a binder has a higher strength compared to a material with only one pozzolanic additive. Other authors [18,31,32,33] have also confirmed that FCCW is another pozzolanic additive that can activate fly ash effectively. FCCW was found to accelerate cement hydration in composite binders with fly ash and initiate pozzolanic reactions faster than fly ash used in isolation.

As FCCW has sufficient activity in the early hydration of cement, this additive may be effective not only in combination with mineral additives with low activity but also with a sufficiently active pozzolanic additive, such as lower-grade metakaolin or metakaolin waste, which often retards cement hydration.

The aim of this study was to analyse the impact of aluminosilicate pozzolanic additives (a spent fluid catalytic cracking catalyst and metakaolin waste from the expanded glass industry) on the cement hydration, binder microstructure formation, and physical and mechanical properties of hardened cement paste.

## 2. Materials and Methods

Portland cement CEM I 42.5 R (PC) produced by Heidelberg Cement (Slite, Sweden) was used for our tests. The chemical composition of the cement is presented in Table 1. The mineral composition of the cement was as follows: C_3_S—56.6%, C_2_S—16.7%, C_3_A—9.0%, C_4_AF—10.6%. The properties of the cement were as follows: a specific surface area of 356 m^2^/kg, a bulk density of 1150 kg/m^3^, compressive values of 28.9 MPa at 7 days and 54.6 MPa at 28 days.

Part of the cement in the mixtures was replaced with the following pozzolanic additives: a spent fluid catalytic cracking catalyst from JSC Orlen Lietuva (Mažeikiai, Lithuania) and metakaolin waste (MK) from an expanded glass manufacturer (JSC Stikloporas, Druskininkai, Lithuania). The chemical compositions of the additives are presented in Table 1. Regarding their chemical composition, FCCW and MK are mainly composed of SiO_2_ and Al_2_O_3_ (91.4% and 88.3%, respectively).

**Figure 1:**
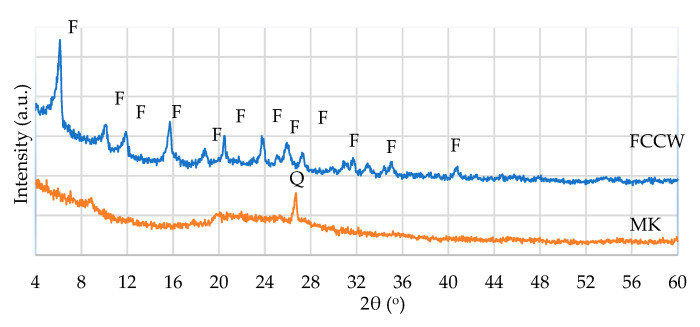
XRD pattern of FCCW and MK: F—faujasite; Q—quartz.

FCCW is a Y-type zeolite that has a crystal structure characteristic of faujasite. MK is an amorphous material with quartz impurities, as identified via XRD spectroscopy (Figure 1).

**Figure 2:**
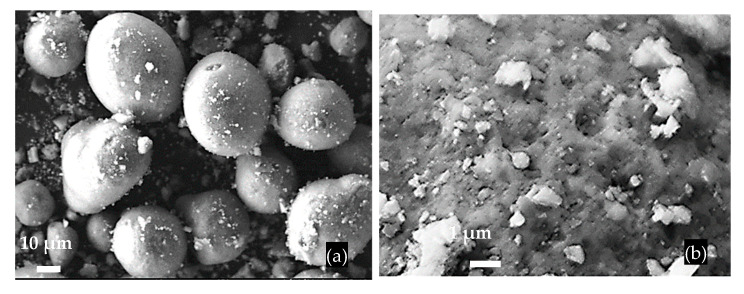
SEM images of FCCW particles (**a**) and the surface of a single particle (**b**).

**Figure 3:**
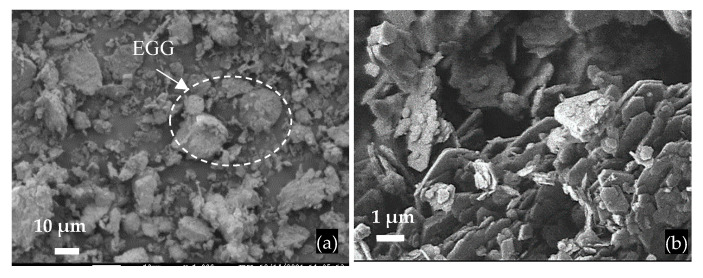
SEM images of MK particles: (**a**) general view of MK with a scrap of expanded glass granules (EGG); (**b**) MK particles.

The FCCW particles have a spherical shape, a diameter of approximately 40 μm, and an uneven surface (Figure 2); the bulk density of FCCW was 945 kg/m^3^. The MK additive used was the mixture of metakaolin and expanded glass scrap (EGG) (Figure 3); the bulk density of the MK we used was 480 kg/m^3^.

The FCCW and MK had good pozzolanic activity values of 1017 mg/g and 1148 mg/g, respectively, determined according to NF P18-513 (newest edition of the document 1 August 2012). This standard describes a saturated lime test, known as the modified Chapelle test. In this method, 1 g of material is reacted with a solution prepared by dissolving 2 g of CaO in 250 mL of deionised water for 16 h at 90 °C. The residual lime was determined by titration with HCl solution. The final result is expressed in mg of Ca(OH)_2_ fixed by g of pozzolan.

The superplasticiser (SP) Melment F10, produced by the German manufacturer BASF (Trostberg, Germany) Construction Polymers, was used. It is a sulphonated melamine formaldehyde condensate in the form of a white powder with a bulk density of 650 kg/m^3^.

Cement pastes with aluminosilicate pozzolanic additives and a water to binder ratio (W/B) of 0.35 and cement pastes of the same composition with 1% added superplasticiser and a W/B of 0.25 were tested (Table 2). The composite pozzolanic additive consisted of FCCW and MK in a 1:1 ratio. The designations of the cement pastes were as follows: C-0, C-CW, C-MK, C-CMK; cement pastes with superplasticiser CP-0, CP-CW, CP-MK, CP-CMK (Table 2). The control samples C-0 and CP-0 did not contain pozzolanic additives.

The cement paste components were mixed in a Hobart mixer for 2 min. After adding the required amount of water, the mixture was mixed for 2 more minutes. Subsequently, the mixture was poured into 160 mm × 40 mm × 40 mm forms and compacted on the vibration table for 10 s. After 24 h, the demoulded specimens were cured in water for up to 28 days at 20 ± 1 °C.

A qualitative analysis of the phase composition of the materials (XRD) was performed on a DRON-7 X-ray diffractometer (Bourevestnik, Saint Petersburg, Russia). A graphite monochromator was used to obtain the X-ray Cu Kα spectrum (λ = 0.1541837 nm). The test parameters were as follows: anode voltage 30 kV; anode current 12 mA; diffraction angle 2θ interval from 5° to 60°, detector step 0.02°; intensity measurement span 2 s. The phases were decoded from the X-ray diffraction patterns using the ICDD diffraction database. Quantitative changes in minerals on the XRD patterns were assessed by the height of the main diffraction peak of the mineral. Anatase was used as an internal standard for the tests in a 9:1 substance/anatase ratio.

Thermogravimetry analysis (TG–DTG) was performed using Linseis STA PT-1600 equipment (Selb, Germany). A platinum crucible with a sample of 60–70 mg was heated in air up to 1000 °C at a heating rate 10 °C/min.

The content of Portlandite (CH) in the hardened cement pastes was calculated from the mass loss recorded on the TG curve in a CH decomposition temperature range of 430–550 °C according to Wang et al. [34] as follows:

CH content (wt %) = Mass loss due to CH dissolution × 74/18(1)

where 74 and 18 are the molar masses of CH and H_2_O, respectively.

The microstructures of the concrete specimens and mineral admixtures were tested using the JEOL JSM-7600F scanning electron microscopy (SEM) device (JEOL, Tokyo, Japan). The following electron microscopy parameters were used: power 10 kV and 20 kV, distance to the samples surface from 6 to 10 mm. Before testing, the splitting surface was coated with a thin electrically conductive layer of gold by evaporating the gold electrode in a vacuum using the QUORUMQ150R ES instrument (Quorum Technologies Ltd., Lewes, UK).

The density values of the samples were calculated based on the mass (0.01 g accuracy) and volume determined based on the dimensions of the samples (0.01 mm accuracy). The compressive strength of the samples after 7 and 28 days was measured using the H200KU hydraulic press (Tinius Olsen, Redhill, UK) according to the EN 1015-11:2007 [35] requirements.

## 3. Results

### 3.1. Microstructure Characteristics

**Figure 4:**
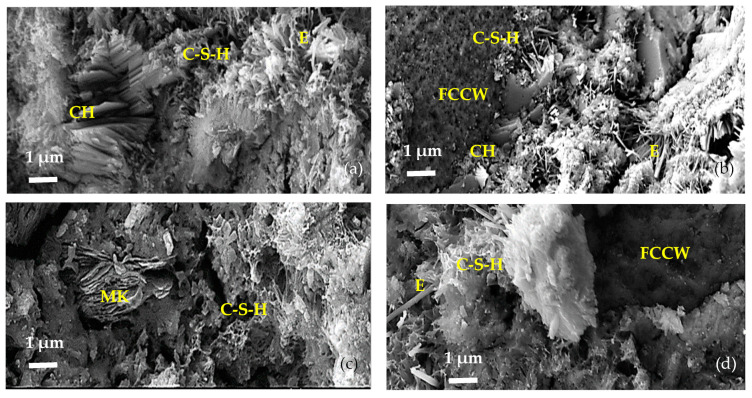
Microstructures of hardened cement pastes composed of C after 7 days: (**a**) C-0, (**b**) C-CW, (**c**) C-MK, and (**d**) C-CMK. CH—portlandite; E—ettringite; C-S-H—calcium silicate hydrate.

SEM images of the microstructures of hardened cement pastes composed of C, taken at day 7 of our study, are presented in Figure 4.

The microstructure of the control sample (C-0, Figure 4a) clearly shows the following hydration products: clusters of platy crystals of portlandite (CH), single needle-shaped crystalline hydrates of ettringite (E), and small particles of amorphous C-S-H [36,37]. In the C-CW composition (Figure 4b), there is a relatively abundant formation of plate-shaped portlandite positioned perpendicularly to the surface of FCCW particle and fine C-S-H interspersed between portlandite crystals. A relatively large amount of amorphous C-S-H phase was observed in compositions containing MK (C-MK, Figure 4c and C-CMK, Figure 4d). No clusters of portlandite crystals were observed on FCCW particles in the compositions with a composite additive (Figure 4d), presumably due to the activating (pozzolanic) effect of MK.

**Figure 5:**
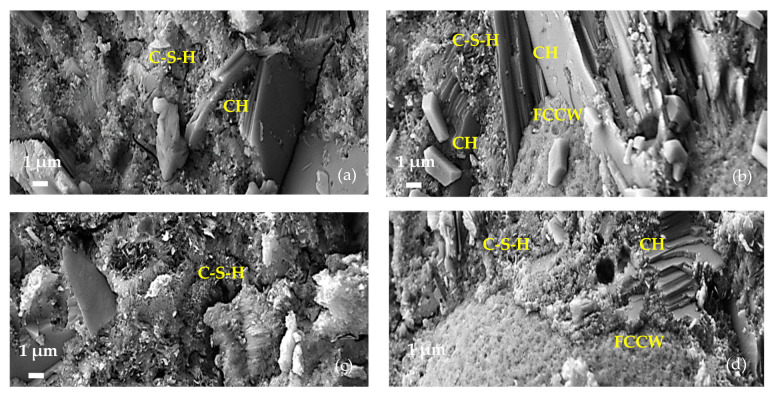
Microstructures of hardened cement paste specimens composed of C after 28 days: (**a**) C-0, (**b**) C-CW, (**c**) C-MK, and (**d**) C-CMK. CH—portlandite; E—ettringite; C-S-H—calcium silicate hydrate.

After 28 days, the microstructures of the samples of all compositions became denser as a result of cement hydration. In the compositions with FCCW, the quantity and size of portlandite clusters on the FCCW surface increased (Figure 5b). In the compositions with a composite additive, the surface of the FCCW particles was covered with a dense C-S-H layer including hydrated portlandite crystals that small in size (Figure 5d). Presumably, the size and amount of portlandite crystals decrease due to pozzolanic reactions, during which CH is depleted by active MK, which is fine and well distributed in the entire cementitious matrix. As a result, more C-S-H and C-A-S-H is formed. It has been shown [38] that the effect of the pozzolanic material commonly occurs between 7 and 28 days, when portlandite is consumed in the reaction.

**Figure 6:**
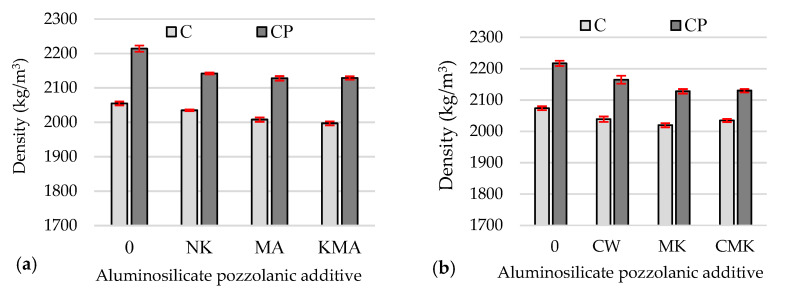
Density values of the hardened cement pastes specimens after 7 days (**a**) and 28 days (**b**).

However, this had no effect on the density of the hardened cement pastes with additives after 28 days of curing (Figure 6b) compared to the density of the samples after 7 days (Figure 6a); in some compositions, it remained practically unchanged, while in others, it increased but only by up to 2%.

At 7 and 28 days, the density values of the hardened samples containing superplasticiser (CP composition) (Figure 6) were 5% to 7% higher than the density values of the samples with a C-based composition due to the reduced W/B ratio. The density values of the control samples with the superplasticiser were 2–4% higher than the density values of the samples with pozzolanic additives due to the lower density of pozzolanic aluminosilicate additives compared to the density of cement and the formation of lower-density hydrates.

**Figure 7:**
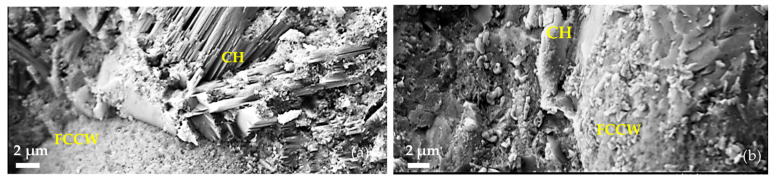
Microstructures of hardened cement pastes with a CP-based composition after 28 days: (**a**) CP-CW; (**b**) CP-CMK.

Similar features to those observed in the microstructures of the samples with a C-based composition were also determined in the CP samples after 28 days (Figure 7): There are evident formations of clusters of portlandite crystals around the spherical FCCW particles in the CP-CW sample (Figure 7a). The interfacial zone between the cement matrix and the FCCW particles is very dense in the compositions with a composite additive, and portlandite crystals were not observed in this zone (Figure 7b).

### 3.2. Analysis of Hydration Products (XRD and TG–DTG)

**Figure 8:**
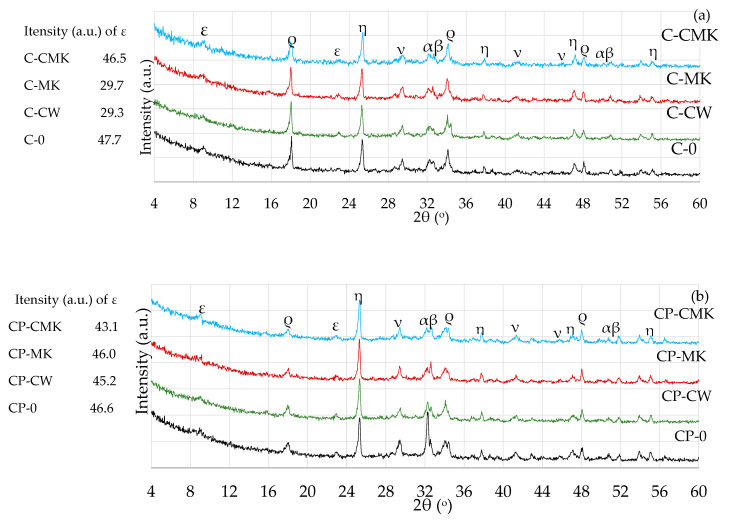
X-ray diffraction curves of hardened cement pastes after 7 days of curing: (**a**) C; (**b**) CP. ε—ettringite; ρ—portlandite; ν—calcite; α—alite; β—belite; η—anatase.

**Figure 9:**
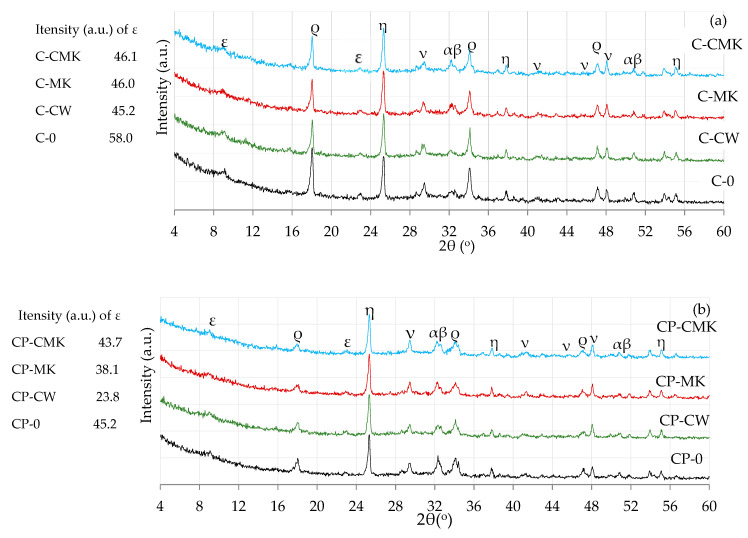
X-ray diffraction curves of hardened cement pastes after 28 days of curing: (**a**) C compositions; (**b**) CP compositions. ε—ettringite; ρ—portlandite; ν—calcite; α—alite; β—belite; η—anatase.

The effect of the aluminosilicate pozzolanic additives on the phase composition of the Portland cement hydration products was determined by using X-ray and thermal analysis methods. The results of the XRD analysis (Figure 8 and Figure 9) showed the presence of the same compounds, namely ettringite (Ca_6_Al_2_(SO_4_)_3_(OH)_12_·26(H_2_O), portlandite (Ca(OH)_2_), and calcite (CaCO_3_), in hardened cement pastes of all compositions after 7 and 28 days of curing.

C-S-H and C-A-S-H, the key products of cement hydration, were not identified in any X-ray diffraction curves irrespective of cement paste composition and curing time (7 and 28 days) due to their amorphous nature [34,39,40,41]. The unreacted cement minerals alite and belite were also identified in the X-ray diffraction curves, as well as anatase, the external standard used for the preparation of the test mixtures. Anatase was used for the calibration of the cement mixture curves in order to compare the intensities of the newly formed components in the different compositions.

It should be noted that lower portlandite peak intensities were obtained for the compositions with aluminosilicate pozzolanic additives (Figure 8 and Figure 9) (card No. 44-1481, the main peak at 34.1°) after being compared to the control specimens. This can be explained by the reduced cement content in the mixture, since the compositions with pozzolanic additives contained 9% less cement than the control compositions, as well as the physical effect (mainly attributed to the additives’ particle filling ability) and the pozzolanic activity of the waste additives [42,43].

A comparison of the portlandite peak intensities of the compositions with aluminosilicate pozzolanic additives only showed that the highest portlandite intensities were recorded in all cases involving compositions with a spent catalyst (C-CW and CP-CW), and the lowest peak intensities were observed in the compositions with a composite pozzolanic additive (C-CMK and CP-CMK). The obtained results could suggest that FCCW not only accelerates early cement hydration [44] but also encourages the formation of hydration products (portlandite) in later cementitious material curing periods. Uniformly distributed fine MK particles in the cementitious material react with portlandite during the pozzolanic reaction to produce products such as C-S-H, C-A-H, and C-A-S-H.

It should be noted that the relative intensity of the portlandite peaks in the CP compositions with the superplasticiser was much lower than in the C compositions (Figure 8a and Figure 9a). According to the authors of [34], this is caused by the superplasticiser’s retarding effect on cement hydration, which subsequently decreases the amount of hydration products. A lower W/B ratio also has an effect on the degree of hydration in CP compositions. Similar findings were confirmed by M. Pereira et al. [40], who observed a lower intensity of portlandite peaks in cement compositions with a lower W/B ratio.

**Figure 10:**
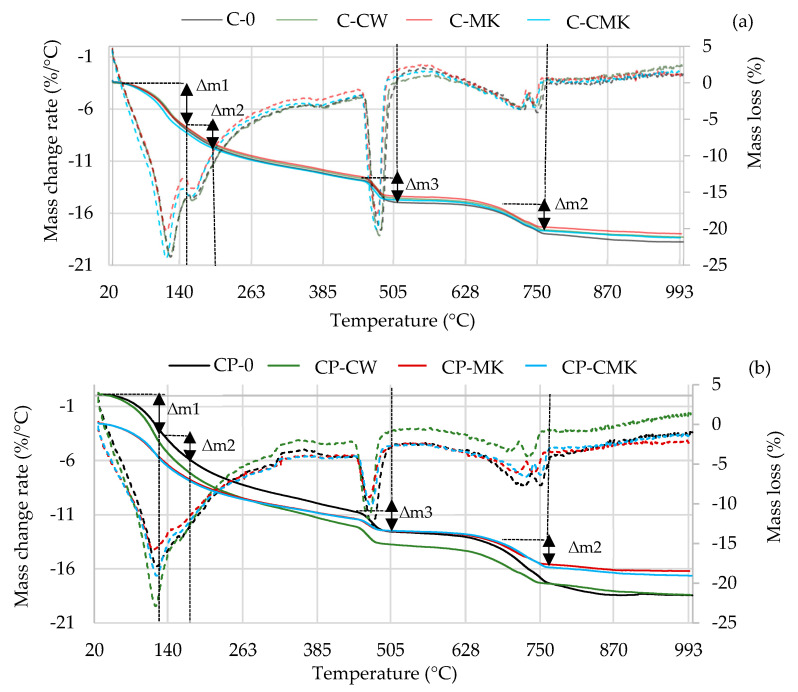
TG–DTG curves of hardened cement pastes after 7 days of curing: (**a**) C compositions; (**b**) CP compositions. (Dashed lines—mass change rate).

**Figure 11:**
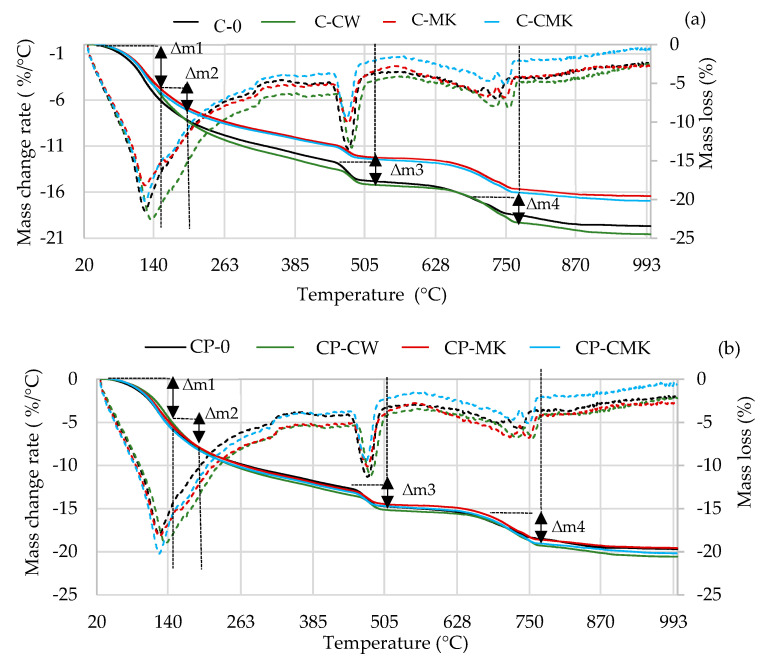
TG–DTG curves of hardened cement pastes after 28 days of curing: (**a**) C compositions; (**b**) CP compositions. (Dashed lines—mass change rate).

Thermogravimetric analysis is a more precise technique than XRD analysis when it comes to determining the amount of portlandite formed in a hardened cement paste. The TG–DTG curves of the cement specimens after 7 and 28 days of curing are presented in Figure 10 and Figure 11.

The TG–DTG curves reveal three endothermic peaks: The first one (30–300 °C) is related to the processes that occur in this temperature range, namely the evolution of free water (at 30–105 °C) and the dehydration of C-S-H (∆m1: at 115–120 °C), ettringite (∆m1: at 100–180 °C), C-A-H, and C-A-S-H (∆m2: at 180–240 °C). The second peak at 430~550 °C (∆m3) is related to portlandite dissolution, and the third peak at 650~750 °C (∆m4) is related to the decarbonisation process [44,45,46,47]. Table 3 shows results pertaining to our evaluation of the portlandite amounts in the temperature range 450–520 °C (determined according to Equation (1)). The results obtained correlate with the XRD analysis results (the intensity of the CH peaks in Figure 8 and Figure 9). At 7 days, the portlandite content in the compositions with aluminosilicate pozzolanic additives was 9.5–13.3%, while in the C and CP compositions, it was 9.3–20.3% lower compared to the control compositions. At 28 days, it was 50% and 20% lower, respectively.

The highest portlandite contents were observed in the compositions containing aluminosilicate pozzolanic additives and in the compositions with FCCW. The lowest amounts of portlandite were observed in the compositions with MK and with a composite pozzolanic additive. Apparently, MK promotes pozzolanic reactions and more portlandite is consumed for the formation of C-S-H and C-A-S-H.

The results of the TG tests show that at 110–350 °C (Table 3), the mass loss in the compositions with aluminosilicate pozzolanic additives plus the superplasticiser was significantly higher compared to the control compositions. These compositions contained 9% less cement, and this could be the reason for the formation of more C-S-H and C-A-S-H phases during the pozzolanic reaction. However, it should be noted that ettringite also decomposes at this temperature, and the ettringite content was found to be higher in the control specimens without the superplasticiser after 28 days, as can be seen in the X-ray diffraction curves, specifically in the intensity of main peak of ettringite (E; card No. 41-1451, the main peak at 9.09° d = 9.67 nm). This is due to the continuing hydration process (Figure 9).

### 3.3. Effect of Aluminosilicate Additives on Mechanical Properties

**Figure 12:**
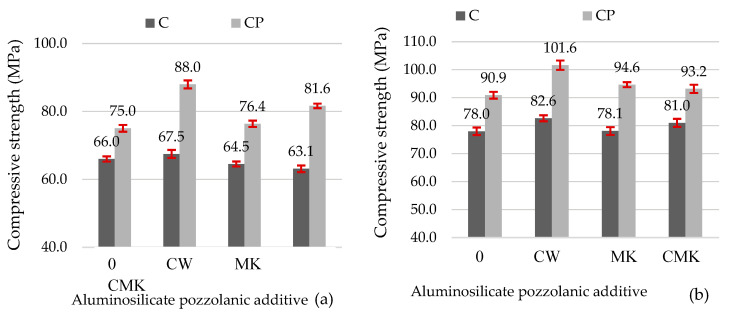
Compressive strength values of the hardened cement pastes after 7 days (**a**) and 28 days (**b**).

Figure 12 illustrates the compressive strength results for the hardened cement pastes. These results indicate that the tested aluminosilicate additives are active pozzolanic materials. When a part of the cement was replaced with these materials, the compressive strength at 28 days remained the same or increased compared to the control samples. Other studies have reported similar results on mechanical properties (for example, the studies conducted by the authors of [16,20], which involved adding FCCW to cementitious materials, and a study conducted by Pundienė et al. involving the addition of metakaolin waste [48]).

The use of the superplasticiser (CP composition) allowed for the production of denser hardened cement pastes with approximately 15% higher compressive strength values compared to the control specimens at both 7 and 28 days (Figure 12) and compared to the control samples with a C-based composition. The compressive strength values of the CP compositions with aluminosilicate pozzolanic additives increased from 15% to 30%.

It should also be noted that all specimens with FCCW (Figure 12) had the highest compressive strengths at 7 and 28 days compared to the samples modified with MK and a composite additive (FCCW+MK). There was little difference in the compressive strength values of the samples with MK and with a composite additive. These results are strongly correlated with the mass losses (Table 3) in the C-S-H and C-A-S-H decomposition temperature range (110–350 °C). The highest mass loss was recorded in the compositions containing FCCW and the superplasticiser (CP-CW). At 7 days, the strength of the C compositions was similar, and at 28 days, the strength values of the control specimens were even lower than the strength values of the specimens with the pozzolanic additives. This can be attributed to the higher levels of formed ettringite in the control samples, as can be seen in the intensity of the main peaks of ettringite in Figure 8a and Figure 9a (ε; Figure 8a and Figure 9a).

## 4. Discussion

In summary, the results of this study show that FCCW, when used to replace a certain percentage of cement in the mixture, adds to the formation of a larger number of platy portlandite crystals. The effect of FCCW on cement hydration is related to its water absorption properties and pozzolanic activity. FCCW particles absorb part of the water when the binder is mixed with water. The reactions that occur during the early hydration period accelerate due to the local decrease in the W/B ratio in the cement paste. Once the material has set, the water accumulated in FCCW particles is used for the further hydration process. The formation of portlandite crystal hydrates was observed around the catalyst particles after 28 days. Therefore, structured elements are formed locally in the structures of hardened cement pastes. The specimens modified with FCCW had higher compressive strength values than the specimens made of cement only due to the higher degree of hydration and pozzolanic activity of FCCW.

Compositions with a composite pozzolanic additive had half of the content of FCCW compared to compositions containing only the FCCW additive; therefore, the effect of FCCW on cement hydration is lower. A change in the structure of the interfacial area between the cement matrix and FCCW particles was observed with a prevalence of C-S-H phase with portlandite inclusions because the active additive MK consumes portlandite for the production of C-S-H and C-A-S-H. This effect was more obvious in the compositions with the superplasticiser.

## 5. Conclusions

1. The aluminosilicate pozzolanic additives (spent fluid catalytic cracking waste and metakaolin waste from the expanded glass industry) investigated in this study were active, and the compressive strength values of the hardened cement pastes after 28 days were the same or higher than those of the samples without pozzolanic additives. The compressive strength values of the samples with the spent fluid catalytic cracking catalyst increased by up to 6% and 12% and by up to 2.5% and 4% when a composite pozzolanic additive (spent fluid catalytic cracking catalyst and metakaolin waste in a 1:1 ratio) was used, respectively, for compositions with and without the superplasticiser, although the cement content of the mixtures was 9% lower. The compressive strengths of the samples with metakaolin waste increased by up to 4% only among the compositions with the superplasticiser.
2. Depending on the pozzolanic additive used, different characteristics regarding the formation of the cement microstructure were observed:
  - The intensive formation of clusters of portlandite crystals around the spent fluid catalytic cracking waste particles were observed after 28 days in the compositions containing 9% of this additive. Such a phenomenon can be explained by the large specific surface areas of the spent fluid catalytic cracking waste particles, which retain the water required for hydration.
  - In the case of the composition without the superplasticiser but with a pozzolanic additive, the portlandite crystals formed on the FCCW particles were smaller in size and had different orientation, and in this composition, a lower content of portlandite was found. In the compositions with the superplasticiser, portlandite was consumed more rapidly for the formation of C-S-H and C-A-S-H due to the pozzolanic effect of MK waste and was no longer identifiable in the FCCW particles in the SEM images, whereas the XRD and DTG results show a reduced amount of portlandite in the entire cementitious paste.

## Figures and Tables

**Table 1:** Chemical composition of PC, FCCW, and MK, (mass, %).

Material	SiO_2_	Al_2_O_3_	Fe_2_O_3_	MgO	K_2_O	Na_2_O	SO_3_	CaO	Mn_2_O_3_	TiO_2_	Cl	LOI
PC	20.4	4.00	3.60	2.40	0.90	0.20	3.10	63.2	–	–	0.05	2.15
FCCW	50.1	41.3	1.30	0.49	0.07	0.20	2.30	0.50	0.06	–	–	1.90
MK	54.3	34.0	1.14	0.51	0.80	3.26	0.15	1.94	–	0.53	–	3.37

**Table 2:** Compositions of cement pastes (mass, %).

Composition	Material				W/B
	PC	FCCW	MK	SP *	
C-0	100	–	–	–	0.35
C-CW	91	9.0	–	–	0.35
C-MK	91	–	9.0	–	0.35
C-CMK	91	4.5	4.5	–	0.35
CP-0	100	–	–	1	0.25
CP-CW	91	9.0	–	1	0.25
CP-MK	91	–	9.0	1	0.25
CP-CMK	91	4.5	4.5	1	0.25

*—the content of components exceeds 100% of the dry mix.

**Table 3:** CH contents in hydrated cement compositions (mass, %) and mass loss in a temperature range spanning from 110 to 350 °C.

Composition	The Amount of Portlandite, wt. %		Mass Loss in the Temperature Range 110–350 °C, %	
	After 7 Days	After 28 Days	After 7 Days	After 28 Days
C-0	12.68	13.90	9.10	11.00
C-CW	11.47	6.96	8.98	9.58
C-MK	11.20	6.74	8.46	9.19
C-CMK	10.99	6.87	8.47	9.29
CP-0	7.60	8.72	8.44	8.71
CP-CW	6.89	7.18	12.78	10.02
CP-MK	6.36	6.74	10.96	9.19
CP-CMK	6.06	6.85	11.96	9.20

## Data Availability

Data are contained within the article.
